# A transcriptome atlas of rabbit revealed by PacBio single-molecule long-read sequencing

**DOI:** 10.1038/s41598-017-08138-z

**Published:** 2017-08-09

**Authors:** Shi-Yi Chen, Feilong Deng, Xianbo Jia, Cao Li, Song-Jia Lai

**Affiliations:** Farm Animal Genetic Resources Exploration and Innovation Key Laboratory of Sichuan Province, Sichuan Agricultural University, Chengdu, 611130 China

## Abstract

It is widely acknowledged that transcriptional diversity largely contributes to biological regulation in eukaryotes. Since the advent of second-generation sequencing technologies, a large number of RNA sequencing studies have considerably improved our understanding of transcriptome complexity. However, it still remains a huge challenge for obtaining full-length transcripts because of difficulties in the short read-based assembly. In the present study we employ PacBio single-molecule long-read sequencing technology for whole-transcriptome profiling in rabbit (*Oryctolagus cuniculus*). We totally obtain 36,186 high-confidence transcripts from 14,474 genic loci, among which more than 23% of genic loci and 66% of isoforms have not been annotated yet within the current reference genome. Furthermore, about 17% of transcripts are computationally revealed to be non-coding RNAs. Up to 24,797 alternative splicing (AS) and 11,184 alternative polyadenylation (APA) events are detected within this *de novo* constructed transcriptome, respectively. The results provide a comprehensive set of reference transcripts and hence contribute to the improved annotation of rabbit genome.

## Introduction

Rabbit (*Oryctolagus cuniculus*) is an important domestic mammal and placed within the family Leporidae of the order Lagomorpha^1^. Due to the closely phylogenetic relatedness to human being, short vital cycle, less aggressive behavior and other advantages, rabbit has been widely used as animal model in biomedical researches^2^. In particular, rabbit resembles human closely with respect to lipoprotein metabolism and is therefore thought to be a preferred animal model for studying human hypercholesterolemia^3^. In addition to the mechanistic investigation of human diseases, rabbit is involved in the experimental studies of surgery^4^. As a monogastric herbivore, rabbit can efficiently utilize plant proteins that are indigestible to human and are economically raised for providing meat, fur and wool products in many parts of the world, especially in China.

Rabbit genome had been sequenced in ~7X depth with the current assembly of 2.66 Gb in size (OryCun2.0)^1^, for which Ensembl genebuild pipeline totally predicted 22,668 genic loci consisting of 24,964 transcripts in Release 85. However, most of the existing gene models are just derived from *in silico* prediction with lack of the reliable annotation on alternative isoforms and untranslated regions, which would prevent accurate evaluation of transcriptome complexity. The improved annotation of genome, therefore, is necessary to facilitate biological researches in rabbit. High-throughput RNA sequencing approach considerably alleviates the genome-wide investigation of gene transcription, alternative isoforms and expression level^5^. However, it has still remained challenging to reliably assemble transcripts in full-length from the short reads, which are essential to characterize the post-transcriptional processes, such as alternative splicing (AS) and alternative polyadenylation (APA) events.

Recently, the single-molecule long-read sequencing technology from Pacific Biosciences (PacBio) provides a better alternative to sequencing full-length cDNA molecules, which has been successively used for whole-transcriptome profiling in human and other species^6–9^. The PacBio long-read sequencing was also specially used for predicting and validating gene models in eukaryotes^10^. In comparison with short read sequencing, the methodological advantages of PacBio RNA sequencing mainly include better completeness to sequence both 5′ and 3′ ends of cDNA molecules, higher accuracy to identify alternative isoforms, and also the increased power to distinguish RNA haplotypes^6, 11^. However, the method of PacBio long-read sequencing could not be directly used to quantify gene expression for the moment. Here, we employ this technology to sequence polyadenylated RNAs of rabbit and provide a transcriptome-wide landscape in relation to gene models and alternative isoforms.

## Results

### PacBio sequencing and error correction of long reads

**Figure 1:**
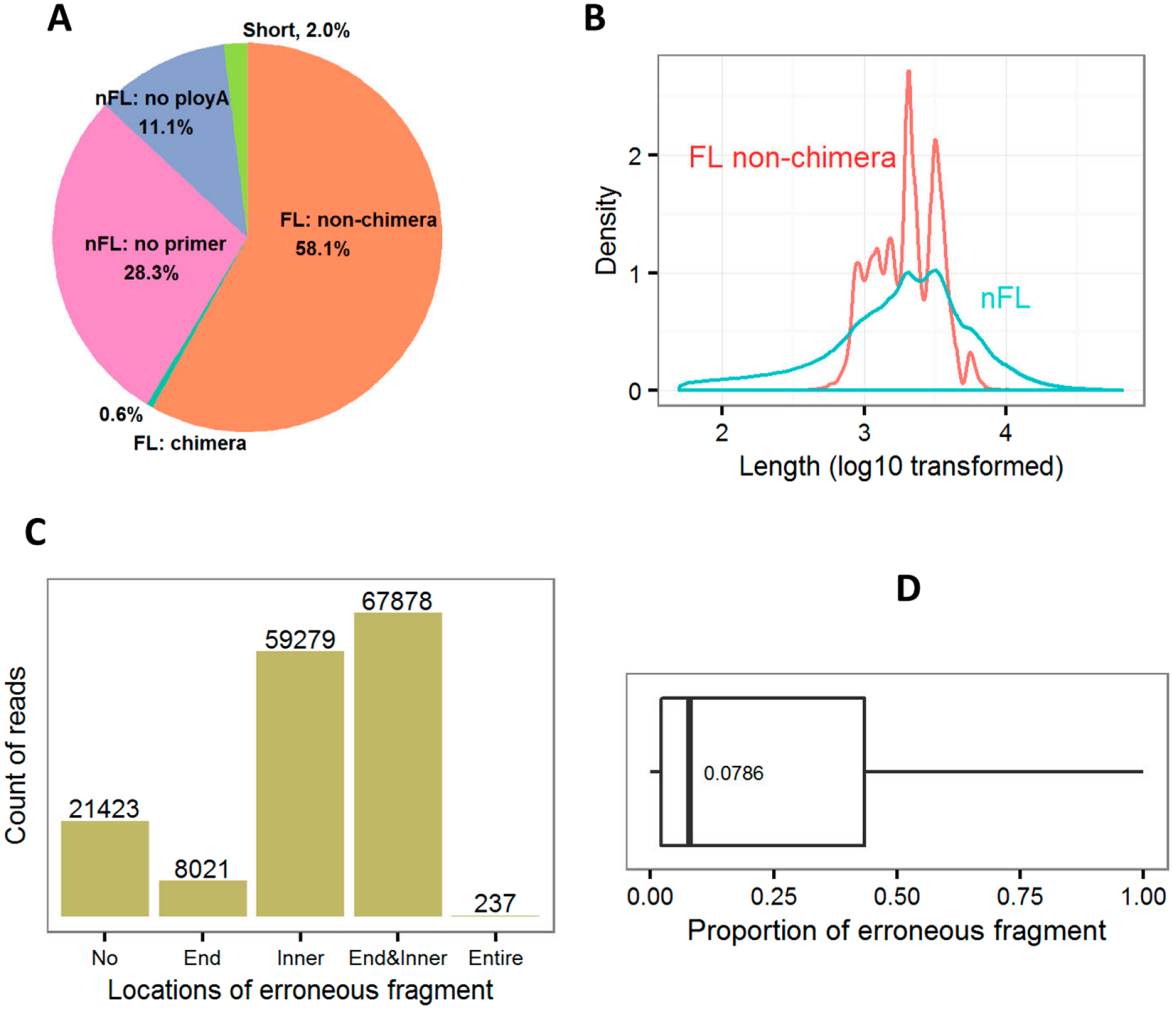
Classification of ROIs and error correction. All ROIs were first classified (**A**) and produced FL and nFL isoform sequences with differential patterns of length distribution (**B**). After the error correction by Illumina short reads, ROIs were categorized according to the occurred locations of erroneous fragments (**C**), including the absence (No), terminal ends (End), inner positions (Inner) and the entire sequences (Entire). Finally, we investigated the length proportion of erroneous fragment relative to raw ROIs (**D**).

From three New Zealand white rabbits at different stages of age, we collected RNA samples of seven organs or tissues and then equally pool them together for library preparation and sequencing. About 61% of zero-mode waveguides, which are referred to as individual sequencing units, were productive among all 13 SMRT cells (Supplementary Table S1). We totally obtained 1,194,102 polymerase reads with full passes > = 0 and the predicted consensus accuracy >0.75, among which 802,358 ROIs (Reads Of Insert) were successfully extracted with mean length of 2,770 bp, quality of 0.92, and 12 passes (Table 1). Length distributions of raw ROIs were consistent with anticipation among the five constructed libraries (Supplementary Figure S1). All ROIs were further classified into 466,034 full-length (FL) non-chimeric and 316,000 non-full-length (nFL) sequences with differential patterns of length distribution (Fig. 1). Based on clustering algorithm of IEC (Iterative Clustering for Error Correction)^12^, we finally got 156,838 consensus isoform sequences.

**Table 1:** PacBio libraries, SMRT cells and sequencing results.

Libraries	SMRT cells	Polymerase reads	ROIs	Mean length	Mean quality	Mean passes
0–1 kb	1	56,113	46,053	1,045	0.95	25.72
1–2 kb	4	357,503	268,241	1,706	0.94	15.42
2–3 kb	3	314,055	214,484	2,484	0.93	9.72
3–6 kb	4	379,372	227,558	3,528	0.91	7.09
5–10 kb	1	87,059	46,022	5,085	0.85	3.00
**Total**	**13**	**1,194,102**	**802,358**	**2,770**	**0.92**	**12.19**

In parallel, we sequenced ~137 million paired-end reads on Illumina platform, from which ~120 million clean reads were generated after quality filtering. These short reads were subsequently used for correcting the consensus isoform sequences of PacBio (Fig. 1), which revealed that a total of 135,178 sequences (86.2%) were deduced to contain erroneous fragment(s) with terminal and/or inner distributions and therefore corrected. However, the length proportions of erroneous fragment were relatively low with the median of 8%. Also, there were 21,423 error-free and 237 completely erroneous isoform sequences, respectively.

### Construction of transcriptomes

The corrected isoform sequences were mapped against reference genome and totally generated 127,037 raw unique alignments. Among them we first filtered out 30,252 potential false alignments using SpliceGrapher^13^, and follow which the sequences with aligned coverage lower than 0.95 were also discarded. A total of 59,051 clean unique alignments were finally retained and clustered into 14,474 independent genic loci. We further removed 22,826 redundant short alignments, which have differences of both <3 bp on intronic coordinates and <15 bp on downstream coordinate of the most 3′ exon. After removal of 39 singleton transcripts, we finally obtained the *de novo* constructed transcriptome that consists of 36,186 transcripts from 14,474 genic loci. Among them 6,418 genic loci (44.3%) had two or more alternative isoforms and 4,164 transcripts (11.5%) were transcripted from single exon (Supplementary Figure S2). For transcripts with multiple exons, median and mean of the most 5′ exons were 140 bp and 201 bp in length, respectively.

Based on Illumina short reads, a total of 46,871 transcripts from 37,850 genes were assembled by the genome-guided method of Cufflinks^14^. Meanwhile, Trinity^15^ *de novo* assembled 571,891 transcripts, 73.8% of which (422,292) were successfully mapped to reference genome.

### Alternative splicing and polyadenylation

**Figure 2:**
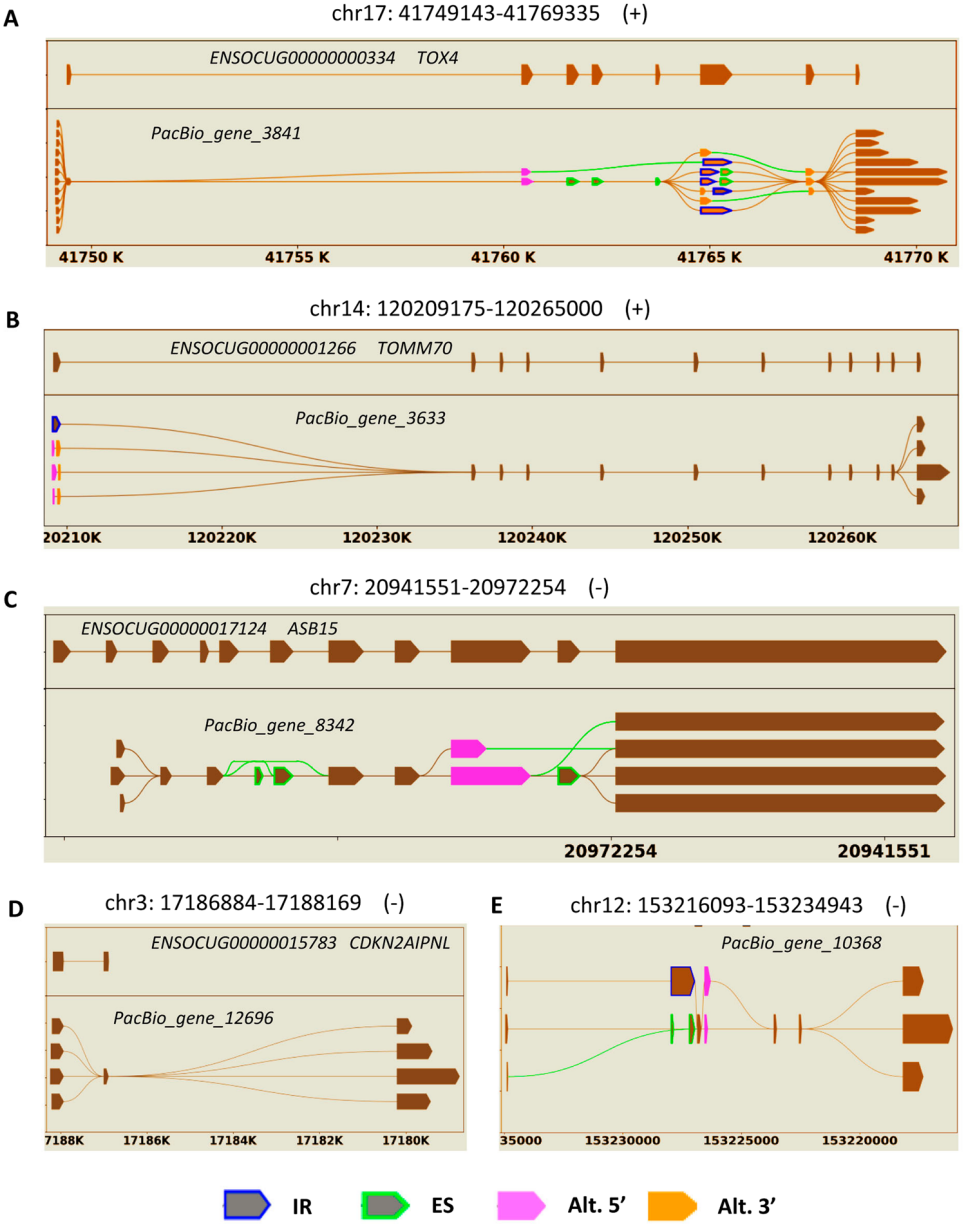
Schematic illustration of alternative isoforms within the PacBio transcriptome. The reference gene models in Ensembl are accordingly placed on upper side and labelled by chromosomal locations, gene ID and name. AS events could be found extensively (**A**) or exclusively detected within terminal exons (**B**). The events of ES and Alt. 5′ are specially shown with the shrunk length of introns (**C**). The newly revealed exon (**D**) and gene (**E**) in reference to Ensembl gene models are also demonstrated.

Within the *de novo* constructed transcriptome by PacBio reads, we totally detected 24,797 AS events (Table 2), including 3,479 intron retention (IR), 7,096 exon skipping (ES), 6,906 alternative 5′ sites (Alt. 5′) and 7,316 alternative 3′ sites (Alt. 3′). By contrast, only 2,398 AS events were observed in total within the reference gene models of rabbit in Ensembl, which was in order of magnitude fewer than PacBio transcriptome. After merging PacBio and Ensembl transcripts together, a total of 34,173 AS events were found with obvious increases for each of all four types. Within PacBio transcriptome we further determined 11,184 APA events at 3,492 genic loci. In Fig. 2, we arbitrarily selected five genes and schematically illustrated various alternative isoforms in comparison with reference gene models in Ensembl. The APA events were also specially exemplified in Supplementary Figure S3.

**Table 2:** Analyses of alternative splicing.

Types	PacBio	Ensembl	Merged
IR	3,479	505	5,066
ES	7,096	766	9,506
Alt. 5′	6,906	463	9,187
Alt. 3′	7,316	664	10,414
**Total**	**24,797**	**2,398**	**34,173**

Among the five examples in Fig. 2, we selected two known (A and C) and one novel (E) genes, all of them have complex AS events as being revealed by PacBio transcripts, and further verified the reliability of alternative isoforms according to mapping evidences of Illumina short reads (Supplementary Figure S4). It was clearly revealed that alternative isoforms of these three genes were reliably supported by the relatively varied coverages of short reads.

### Comparison with Ensembl annotation

After being compared with reference annotation of rabbit genome in Ensembl, a total of 3,334 genic loci consisting of 3,637 transcripts within PacBio transcriptome had not been annotated yet and were believed to be novel. Furthermore, there were 12,112 transcripts having identical intronic coordinates with reference annotations. On the whole, these novel transcripts showed shorter length with the predominant distribution between 1,000 and 2,000 bp (Supplementary Figure S5).

### Classification of non-coding RNAs

**Figure 3:**
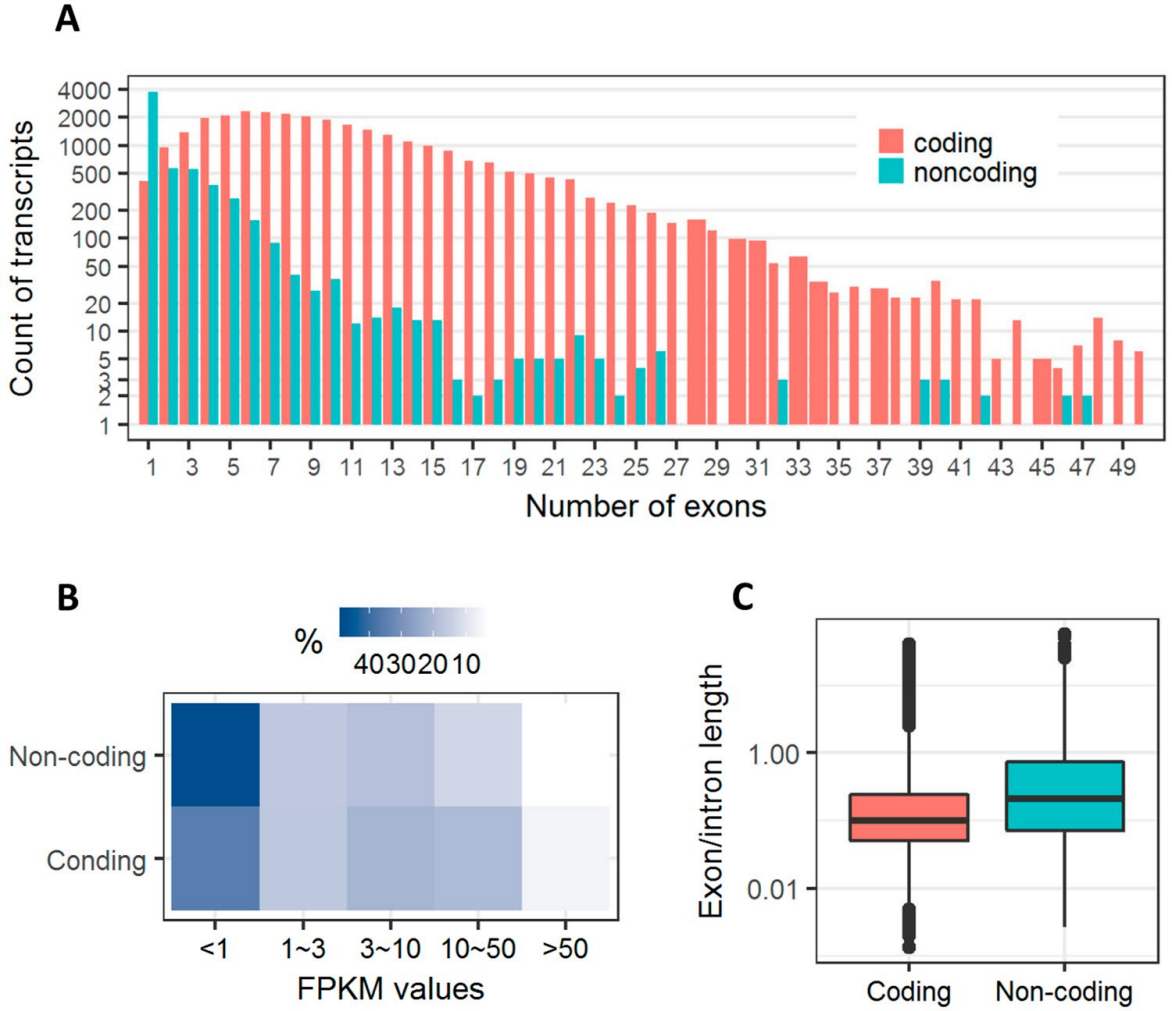
Comparisons between protein-coding and non-coding transcripts for the number of exons (**A**), expression levels (**B**) and length ratio of exon to intron (**C**). FPKM, fragments per transcript kilobase per million fragments mapped.

According to homologous searches against reference protein database, 30,183 and 6,003 transcripts were believed to be the protein-coding and non-coding RNAs, respectively. We additionally observed that non-coding transcripts have less exons, lower expression levels, and slightly higher ratio of exon to intron in length than that of protein-coding transcripts (Fig. 3). Genomic distributions of non-coding transcripts were also classified into 1,794 intergenic and 3,558 genic locations with respective to the protein-coding transcripts that were newly detected in the present study and already annotated within reference genome in Ensembl (Table 3); the two classes could be further divided into different sub-classes according to the recently proposed definitions by Wucher and colleagues^16^.

**Table 3:** Classification of non-coding transcripts.

Intergenic class						Genic class						Others
Divergent		Convergent		Same strand		Overlapping		Containing		Nested		651
U	D	U	D	U	D	E	I	E	I	E	I	
349	0	0	282	871	292	1,062	32	281	16	1,299	868	

U, upstream; D, downstream; E, exonic; I, intronic. Definitions for different classes and sub-classes were described in the initial reference.

### Recovery of paralogous genes

**Figure 4:**
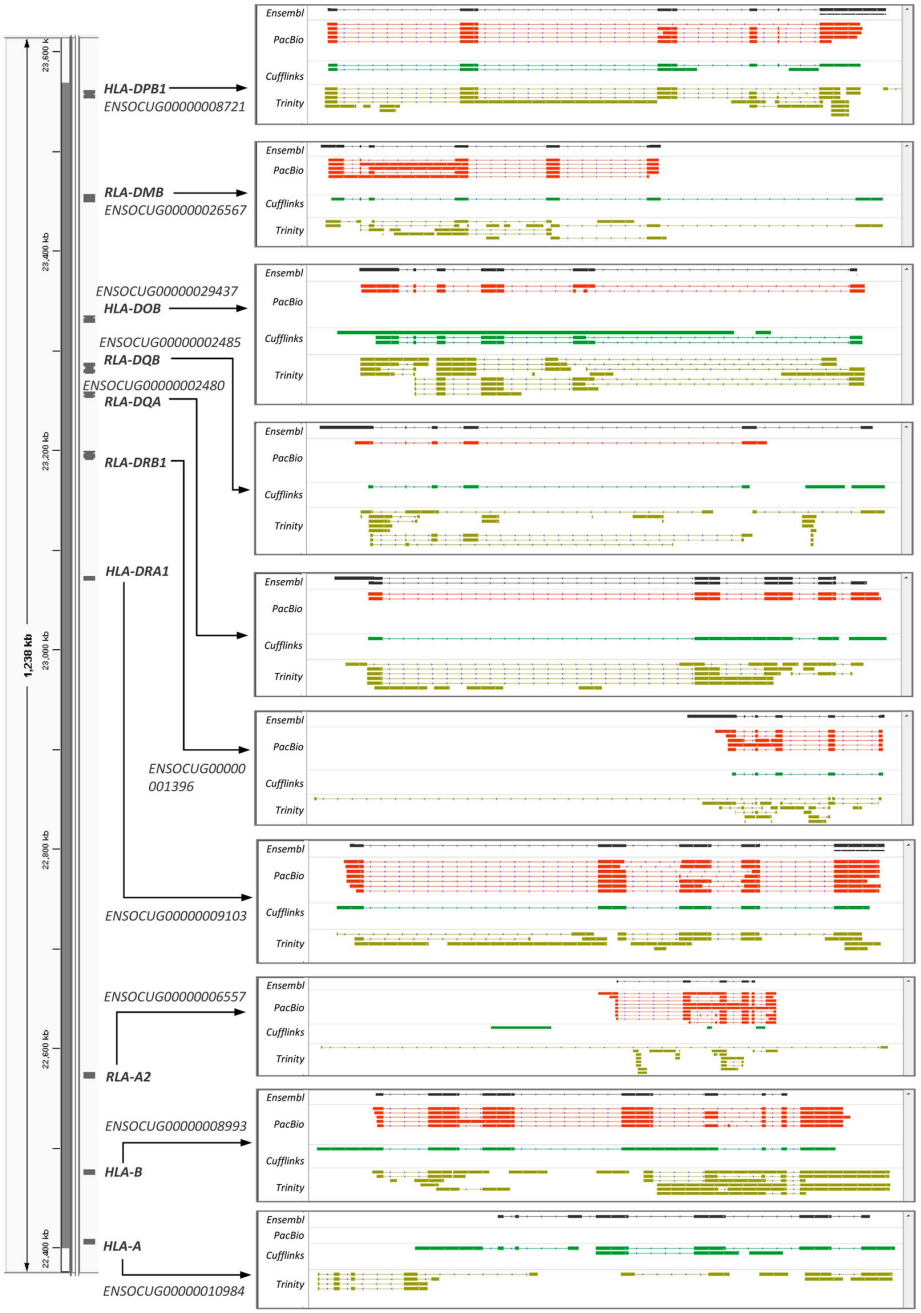
Recovery of ten MHC genes by PacBio transcripts and the assembled transcripts from short reads. Chromosomal locations, name and Ensembl accession number for each gene are shown on the left. The exon-intron structures are illustrated into the separate boxes and within each of them the reference transcripts in Ensembl (Black), PacBio transcripts (Red), and the assembled transcripts by Cufflinks (Green) and Trinity (Olive) are listed in order from top to bottom.

We selected ten Major Histocompatibility Complex (MHC) paralogous genes, all of which are adjacently distributed within a 1.2-Mbp region on chromosome 12 (Fig. 4), to exemplify the power of PacBio long-read sequencing to identify the paralogues. With an exception of *HLA-A*, all gene structures were well recovered by PacBio transcripts in comparison with reference annotation. Moreover, PacBio transcripts also supported many novel isoforms that have not been annotated yet. By contrast, performances of the assembled transcripts from short reads by genome-guided method were obviously lower than PacBio transcripts in terms of both the recovery of gene structure and number of isoforms. All of these paralogous genes were poorly assembled using the *de novo* approach, which was very apt to produce the fragmented and confusing transcripts.

## Discussion

After the initial domestication occurred in southern France ~1,400 years ago^1^, modern rabbits are now raised for laboratory animal, meat, fur and wool throughout the world. In comparison with other domestic animals such as pig^17^ and cattle^18^, little attention has been paid to genomic biology of rabbit despite the fact that the draft genome was early sequenced and publicly available in 2009. Recently, an international project titled “LaGomiCs” has been proposed for sequencing genomes of all species within the order Lagomorpha, which is expected to considerably increase our understanding of important biological problems in this and related taxonomic clades^19^. In the present study, we employed PacBio single-molecule long-read sequencing technology for whole-transcriptome profiling in rabbit and generated a large number of gene models and alternative isoforms that have not been annotated yet. The results therefore expand the reference set of gene transcriptions in rabbit. Of course, this profiling of rabbit transcriptome would not be exhaustive mainly because the limited biological samples were sequenced. Because our aim is to provide a general encyclopedia of gene transcriptions, we therefore sequenced the pooled RNA samples from different organs or tissues together with the reduced sequencing cost.

The methodological strengths of PacBio RNA sequencing were comprehensively investigated in human^6^, which had been proposed to be superior to methods of short read sequencing mainly due to the advantage for obtaining full-length transcripts. Furthermore, the accuracy of PacBio transcripts for identifying AS and APA events had been experimentally verified^8, 9^. In the present study, we also showed the obviously improved power of PacBio transcripts for recovering the highly homologous sequences among ten MHC genes than the assembled transcripts from short reads. The length distribution of the most 5′ exons of our PacBio transcripts is consistent with former report in human^6^, which would indicate the comparable sequencing completeness in rabbit.

Human transcriptome was revealed to be much more complex than previously believed due to the application of PacBio long-read sequencing technology, which produced a bulk of novel isoforms that had not yet been annotated^6^. In a similar study for sequencing chicken transcriptome, ~10,000 novel isoforms and genic loci were also found to be absent from reference annotation^7^. For our newly sequenced transcriptome in rabbit, ~33,000 isoforms were transcripted from known genic loci and most of them have not been included within the reference annotation in Ensembl. Additionally, more than 3,000 genic loci were found to be novel. Because we designed strict criteria (see Materials and Methods) for filtering the genomic alignments of PacBio reads to reduce false positives, the actual complexity of rabbit transcriptome would be higher than the current observation.

About 17% of all newly generated transcripts in the present study were deduced to be non-coding RNAs, which is similar to the former study^6^. However, the occurrences of false positives of non-coding transcripts could not be absolutely excluded because this conclusion just depends on the computational approach of homologous search against reference protein database. Derrien and colleagues^20^ reported that the long non-coding RNAs in human are characterized by less exons and lower expression than protein-coding RNAs, both of which were also detected in the present study. However, we observed a relatively higher proportion of single-exon transcripts among non-coding RNAs than that in human^20^, for which one possible explanation is that many low-expressed multiple-exon RNAs could not be detected because of the limited sequencing depth of PacBio technology. Furthermore, it would also result into a slight difference of exon/intron length between protein-coding and non-coding RNAs observed in the present study.

In contrast to the merit for producing longer reads, PacBio long-read sequencing technology is also characterized by the relatively high error rate^21^, for which, therefore, many computational approaches have been proposed for error correction in aid of the short reads^22, 23^. Accordingly, we sequenced the short reads in parallel to correct PacBio reads; and analysis results also revealed that more than 80% of PacBio reads contain erroneous fragments more or less. Therefore, it is suggested that the prior error correction of PacBio reads is necessary.

## Materials and Methods

### Ethics statement

The study design was approved and all methods were performed in accordance with guidelines of Institutional Animal Care and Use Committee in College of Animal Science and Technology, Sichuan Agricultural University.

### Collection of samples and RNA preparation

We collected three healthy female New Zealand white rabbits at 21, 49 and 84 days of age, respectively. Seven organs or tissues consisting of brain, heart, lung, liver, spleen, skeletal muscle from hind leg, and intestinal *sacculus rotundus* were sampled for each rabbit. Subsequently, all 21 samples were subjected to RNA extraction using RNAiso Pure RNA Isolation Kit (TaKaRa, Japan), which was followed by treatment of DNaseI. NanoVue Plus was used to assess concentration and quality of the extracted RNAs. Finally, one microgram for each RNA sample was equally pooled together and subjected to PacBio single-molecule long-read sequencing (Pacific Bioscience, Menlo Park, USA) and Illumina PE150 sequencing (Illumina, San Diego, USA) in parallel.

### Library construction and PacBio sequencing

According to the official protocol, one microgram of RNA was reversely transcribed using Clontech SMARTer cDNA synthesis kit. After PCR amplification, quality control and purification, we performed size selection using BluePippin Size Selection System protocol and herein produced five libraries corresponding to fragments of 0–1, 1–2, 2–3, 3–6 and 5–10 kb in length, respectively. The cDNA products were then subjected to construction of SMRTbell Template libraries using SMRTBell Template Prep Kit. Finally, a total of 13 SMRT cells were sequenced on PacBio RS II platform using P6-C4 chemistry with 4–6 h movies.

### Illumina short-read sequencing and transcriptome assembly

The Illumina library was prepared using NEBNext UltraTM RNA Library Prep Kit (E7530L) for Illumina (NEB, USA). Briefly, polyadenylated RNA was isolated and fragmented into ~200 bp fragments. The first-strand cDNA was synthesized using random hexamer-primers, which was followed by synthesis of the second strand. The purified and repaired double-stranded cDNA fragments were selected by size. The amplified mRNA libraries were finally sequenced on Illumina HiSeq X Ten platform for generating 150 bp paired-end reads. Raw short reads were subjected to quality filtering using NGS QC Toolkit v2.3.3^24^, for which we trimmed the first five bases from the 5′ end of read and removed reads consisting of the low quality bases (QA ≤ 30) >20% or ambiguous bases >1%.

**Figure 5:**
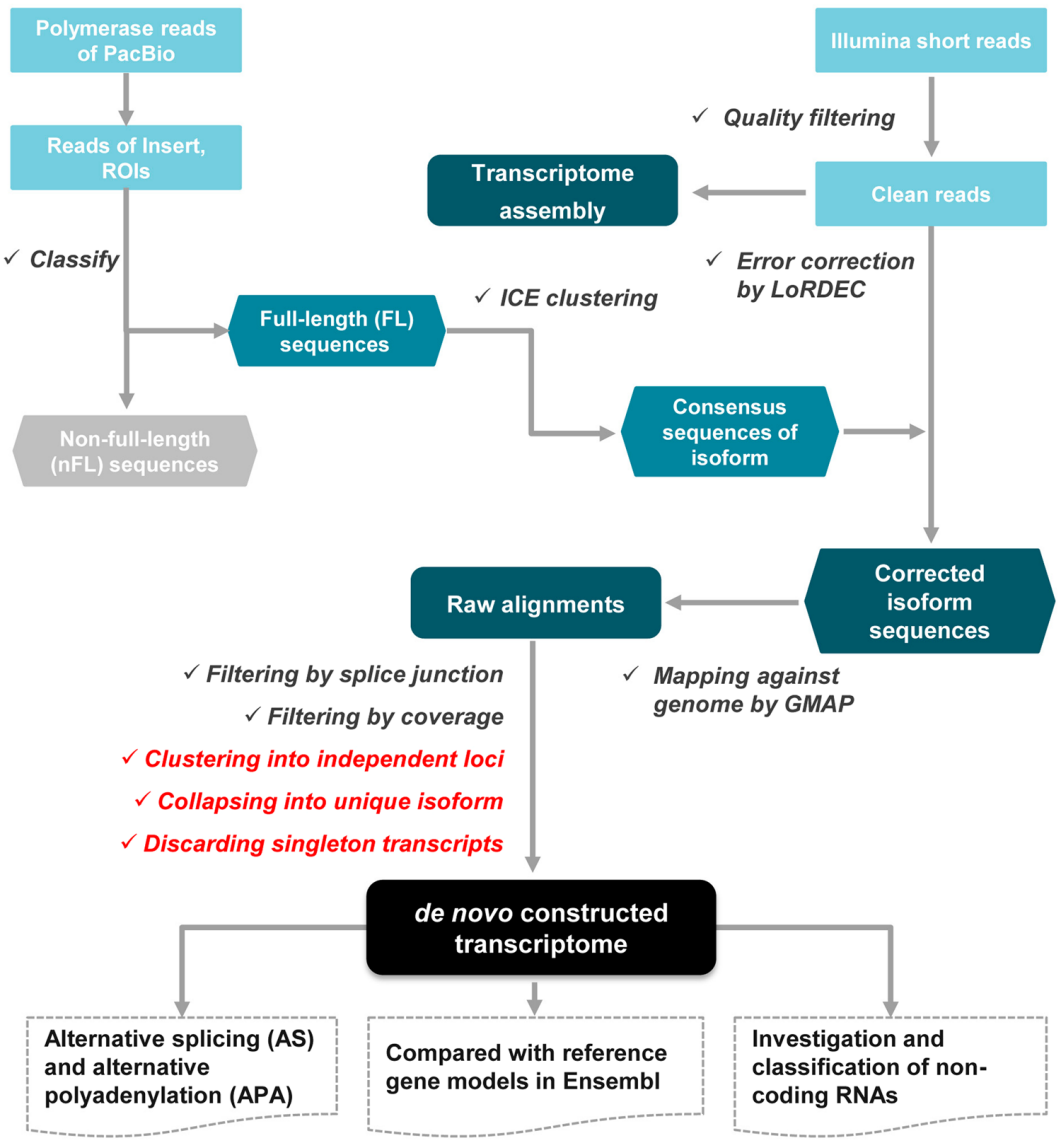
Pipeline of bioinformatic analyses. The processing steps are individually ticked, and among which the clustering, collapsing and filtering of GMAP alignments were conducted using our in-house scripts (in red).

The whole pipeline of bioinformatic analyses involved in the present study is outlined in Fig. 5. In addition to correction of PacBio reads, Illumina short reads are used for independently assembling transcripts by tools of Cufflinks v2.2.1^14^ and Trinity v2.3.0^15^, respectively. Briefly, all clean short reads were first mapped to reference genome of rabbit using Tophat2 v2.1.1^25^ with the following parameters: alignment with no more than 3 mismatches (−N 3), no more than 2 gaps (–read-gap-length 2) and no more than 4 edit distances (–read-edit-dist 4). Subsequently, we assembled transcripts using Cufflinks with the default parameters^14^. With the default parameters, transcripts were *de novo* assembled using Trinity^15^ and further mapped against reference genome using Blat v35^26^.

### Error correction of PacBio reads

According to the official protocol, raw Polymerase reads that have full passes > = 0 and the predicted consensus accuracy >0.75 were selected for producing ROIs. After the ROIs shorter than 50 bp in length were discarded, they were classified into FL and nFL transcript sequences according to whether 5′/3′ cDNA primers and poly(A) tail were simultaneously observed or not. We subsequently employed three strategies for improving accuracy of PacBio reads. First, only FL ROIs were selected due to the relatively high reliability. Second, all sequences were subjected to isoform-level clustering by IEC algorithm and herein produced consensus sequences of isoform^12^. Finally, the isoform sequences were corrected in aid of Illumina short reads using LoRDEC tool v0.6 with -k 21, -s 3, and default setting for other parameters^23^.

### Mapping and transcriptome construction by PacBio reads

The corrected isoform sequences were aligned against reference genome using GMAP aligner v2016-08-24^27^ with the parameters: –min-identity 0.95 and –allow-close-indels 2. The sequences with multiple and chimeric alignments were excluded from the following analyses. Among raw unique alignments, we first filtered out false splice junctions using SpliceGrapher v0.2.4^13^, in which the models of splice sites were designed as donors of GT, GC or AT, and acceptor of AG or AC, respectively. The filtered alignments were clustered into independent genic loci such that every locus consists of the overlapped sequences with each other. Within each locus, alignments that have same coordinates on both intronic boundaries and 3′ end of the last exon were collapsed into unique sequence and the longest transcript was selected as representative of this isoform. For locus having multiple isoforms we also discarded the singleton transcript, which is defined that a transcript doesn’t have any splice junction being shared with others. When all sequences within a locus are singleton transcripts, only the longest transcript is retained. Here, we got the high-confidence alignments of isoform sequences and by which the full transcriptome was successfully *de novo* constructed.

### Transcriptome analyses

For the *de novo* constructed transcriptome by PacBio reads, transcriptome-wide AS events were analyzed using SpliceGrapher^13^, which predicts four AS types consisting of IR, ES, Alt. 5′ and Alt. 3′. Among them, IR is inferred that one intron is retained within a longer exon and simultaneously flanked by two shorter exons. ES is defined when an exon is absent in some transcripts but present in others. When an intron is excised at more than one sites and linked to its 5′ or 3′ exons with different boundaries, they are considered as the Alt. 5′ and Alt. 3′, respectively. We further detected occurrence of APA when the aligned transcripts have same intronic coordinates but differ in downstream coordinate of the last exon with >15 bp in length.

The newly constructed transcriptome was first compared with reference annotation of rabbit genome in Ensembl (Release 85). The PacBio transcripts are classified as derivation from the known genes if the aligned strand and chromosomal locations overlap with any already annotated gene model; otherwise the sequences are transcribed from novel genic loci. For sequences being transcripted from known gene, a transcript was further defined as novel isoform if its intronic coordinates could not overlap any of the existing reference transcripts.

We further distinguished all PacBio transcripts between protein-coding and non-coding RNAs using dbHT-Trans tool v1.0^28^, which deduces all potential open reading frames of sequence and subsequently translate into amino acids for homologous search against reference database. If a transcript could computationally encode protein that has one or more known homologs, it is regarded as protein-coding RNA and vice-versa. In the present study, we searched against the reference proteomes of rabbit, mouse, rat and human deposited in Uniprot database (release 2016_09), in which a positive hit was defined that at least 50% fragment of query sequence has more than 70% identity with target sequence. Subsequently, FEELnc tool v12/07/2016 was employed for classifying non-coding RNAs with the default parameters, which compares the genomic location and transcription orientation with respect to the nearest protein-coding RNA^16^. Two intrinsic features of both the number of exons per transcript and length ratio of exon to intron were compared between protein-coding and non-coding RNAs using in-house scripts. Furthermore, we employed RSEM tool v1.1.11 for quantifying transcript abundances by Illumina short reads with the default parameters^29^, which outputs the measure of FPKM (fragments per transcript kilobase per million fragments mapped).

### Data availability

The newly constructed rabbit transcriptome (in GTF file) by PacBio reads in the present study is available upon request.

## Electronic supplementary material

Supplementary Data
