# Supplementary material for: A transcriptome atlas of rabbit revealed by PacBio single-molecule long-read sequencing
Source: Sci Rep. 2017 Aug 9;7:7648. doi: 10.1038/s41598-017-08138-z (PMC5550469; doi:10.1038/s41598-017-08138-z)
Supplement: Supplementary file 1 — Supplementary Data [file 41598_2017_8138_MOESM1_ESM.pdf]

Suppementary Data

A transcriptome atlas of rabbit revealed by PacBio single-molecule long-read sequencing

Shi-Yi Chen, Feilong Deng, Xianbo Jia, Cao Li, Song-Jia Lai

Supplementary Table S1 Productivity of the zero-mode waveguide (ZMW)

Libraries	SMRT Cell ID	Productive ZMWs	Productivity 0 (%)	Productivity 1 (%)	Productivity 2 (%)
0-1K	1	150,292	58.14%	37.34%	4.52%
1-2K	2	150,292	50.08%	43.85%	6.07%
1-2K	3	150,292	2.75%	61.97%	35.28%
1-2K	4	150,292	30.52%	60.66%	8.82%
1-2K	5	150,292	20.59%	71.41%	8.00%
2-3K	6	150,292	18.72%	72.29%	8.99%
2-3K	7	150,292	13.72%	67.08%	19.20%
2-3K	8	150,292	6.53%	69.60%	23.87%
3-6K	9	150,292	3.40%	67.05%	29.55%
3-6K	10	150,292	6.57%	62.11%	31.32%
3-6K	11	150,292	33.19%	54.29%	12.52%
3-6K	12	150,292	24.59%	68.98%	6.43%
5-10K	13	150,292	12.59%	57.93%	29.48%

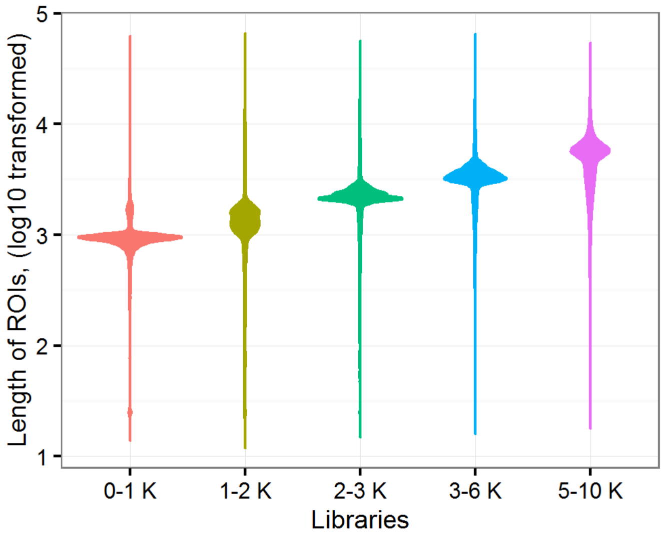

Supplementary Figure S1. Violin plots show the length distribution of raw ROIs among the five sequenced libraries.

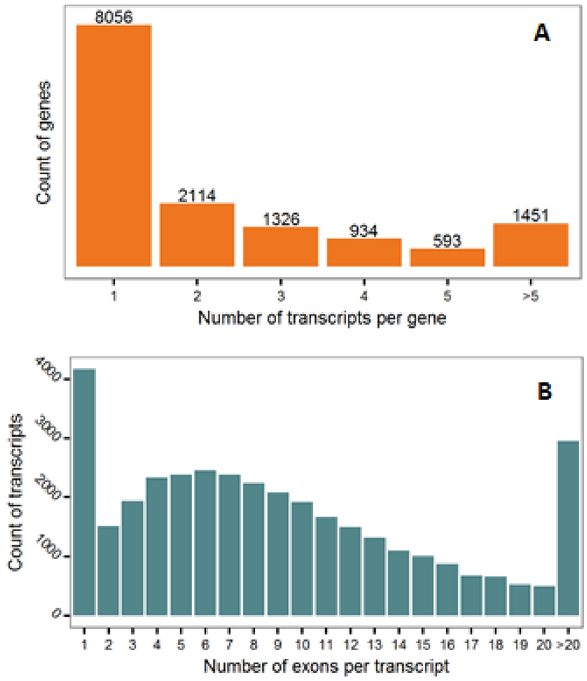

Supplementary Figure S2. Numbers of transcripts per each gene (A) and exons per each transcript (B) for the *de novo* constructed transcriptome of PacBio reads.

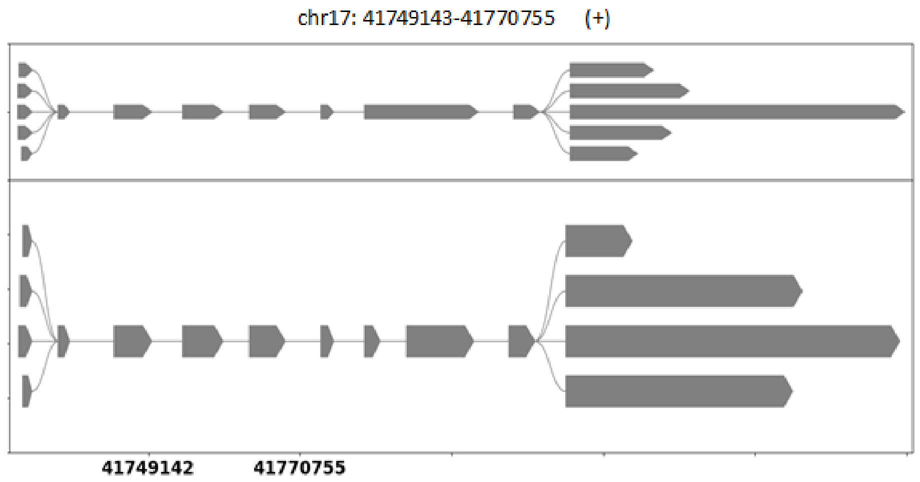

Supplementary Figure S3. Schematic illustration of alternative polyadenylation. This gene is also shown in Figure 2A, which is further used here for demonstrating nine APA events as being observed in two isoforms. The intron lengths shrink for better illustration.

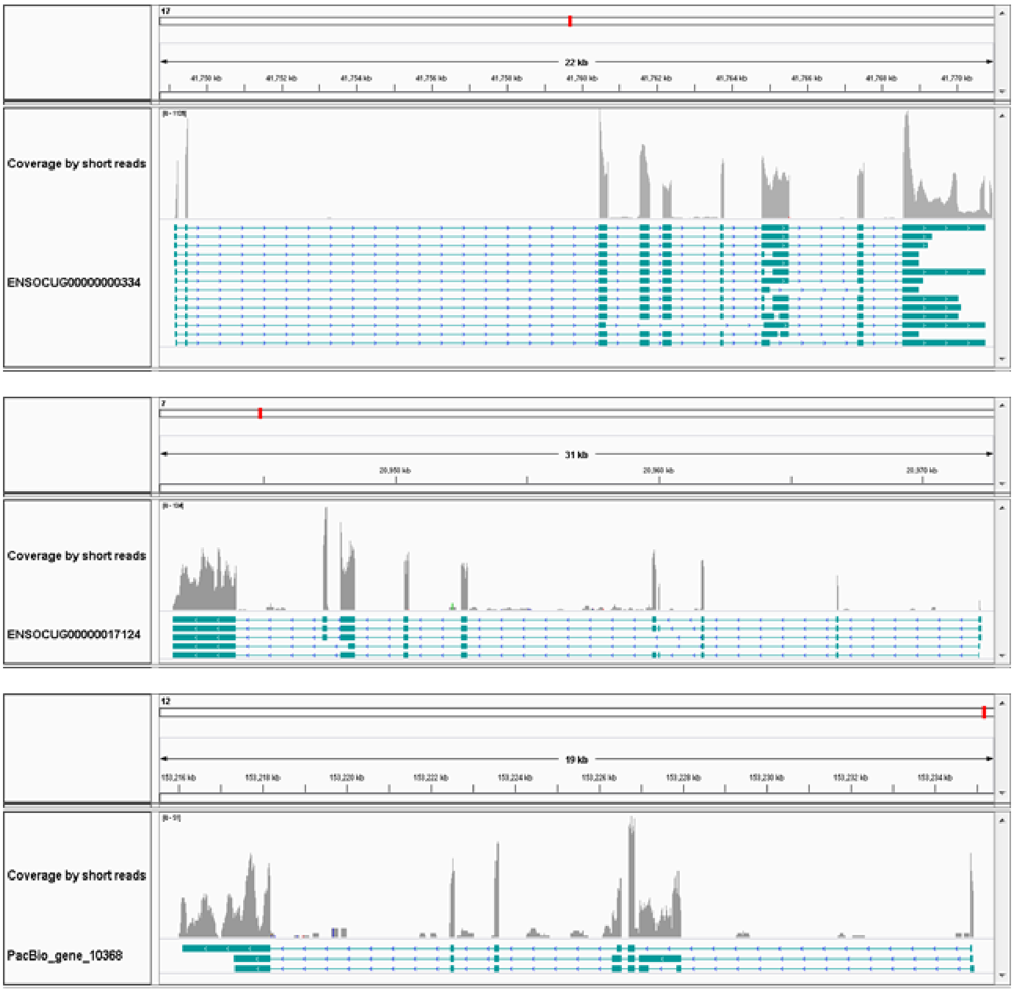

Supplementary Figure S4. Comparison between the gene structures of PacBio transcripts (Blue) and mapping coverages of Illumina short reads (Grey). Chromosomal locations and other information for these three genes are shown in Figure 2A, 2C and 2E, respectively.

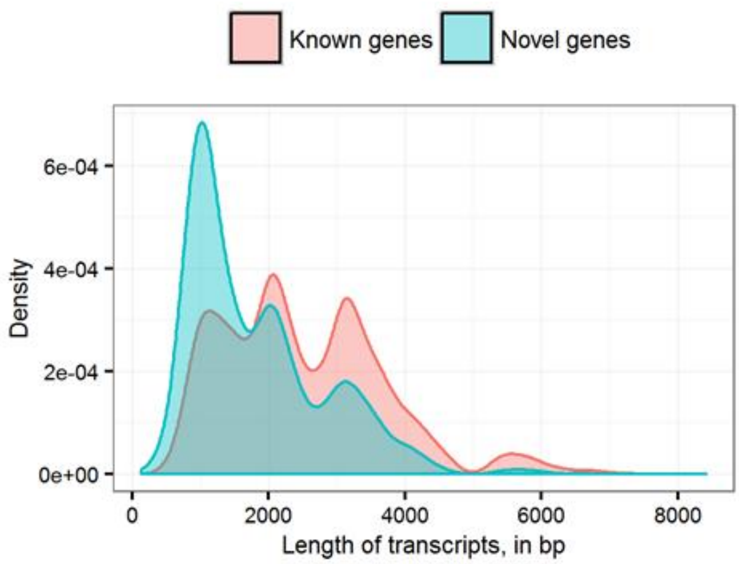

Supplementary Figure S5. Length distribution of PacBio transcripts derived from known and novel genic loci.
